# Long-term clinical and cost-effectiveness of a fully automated digital cognitive behavioural therapy for insomnia: 2-year follow-up of a single-blind, superiority, randomised controlled trial

**DOI:** 10.1016/j.lanepe.2026.101691

**Published:** 2026-05-08

**Authors:** Daniel Vethe, Zareen A. Khan, Cecilie L. Vestergaard, Vidar Halsteinli, Simen B. Saksvik, Øystein Vedaa, Børge Sivertsen, Gunnar Morken, Knut Langsrud, Lee M. Ritterband, Otto Robert F Smith, Jan Scott, Håvard Kallestad

**Affiliations:** a Clinic of Mental Healthcare, St. Olav’s Hospital, Trondheim University Hospital, Trondheim, Norway; b Department of Mental Health, Norwegian University of Science and Technology, Trondheim, Norway; c Department of Health Promotion, Norwegian Institute of Public Health, Bergen, Norway; d Department of Research and Innovation, Helse Fonna HF, Haugesund, Norway; e Institute of Neuroscience, Newcastle University, Newcastle upon Tyne, UK; f Department of Psychiatry and Neurobehavioral Sciences, Center for Behavioral Health and Technology, University of Virginia, Charlottesville, VA, USA; g Centre for Evaluation of Public Health Measures, Norwegian Institute of Public Health, Oslo, Norway; h Department of Teacher Education, NLA University College, Bergen, Norway

**Keywords:** Insomnia, dCBT-I, Digital therapy, Cost-effectiveness, Long-term effectiveness

## Abstract

**Background:**

We evaluated the long-term clinical and cost-effectiveness of fully automated digital cognitive behavioural therapy for insomnia (dCBT-I) or online patient education (PE) in the Norwegian general population.

**Methods:**

A parallel-group, participant-blinded, superiority randomised controlled trial in self-referred adults with significant insomnia symptoms (Insomnia Severity Index [ISI] ≥12). Participants completed automated screening prior to randomisation and outcomes were assessed over 2 years. The primary outcome (9-week clinical effectiveness) has been published. We now report intention-to-treat analyses from the 6-month and 2-year follow-ups. Incremental cost-effectiveness ratio (ICER), with non-parametric bootstrapping, summarized the between-group societal costs in 2019 Euros, and quality-adjusted life years (QALYs). The trial was preregistered at ClinicalTrials.gov (NCT02558647) and followed a prespecified protocol.

**Findings:**

1720 participants (1167 [67.8%] female; mean age 44.4) were randomised between February 26, 2016, and July 1, 2018 (867 to dCBT-I vs. 853 to PE). The final follow-up included 587/1720 (34.1%) participants (dCBT-I = 315; PE = 272), a median of 28.3 months (IQR 22.6 to 34.0) after baseline. At 2-year follow-up the mean ISI was 10.7 (SD 5.9) in the dCBT-I group and 13.4 (5.9) in the PE group (estimated difference: −1.77 [95% CI: −2.65 to −0.90]; Cohen’s *d*= −0.43). The dCBT-I group reported −€278 [95% CI: −1413 to 858] lower costs and 0.025 QALYs gained [95% CI: 0.010 to 0.041] compared with PE, yielding a 94% probability of cost-effectiveness at €0 willingness-to-pay (ICER: −€10,973 per QALY gained). No harms or adverse events were reported.

**Interpretation:**

Compared with online PE, fully automated, low-threshold dCBT-I demonstrates greater short-term (9 week) and longer-term improvements (≥2 years) in insomnia severity and is likely cost-effective over the 2-year horizon. However, effects attenuated over time and long-term certainty is limited by attrition.

**Funding:**

Norwegian Council of Research; Liaison Committee for Education, Research and Innovation in Central Norway.

**Research in context**

**Evidence before this study**

We searched PubMed, Embase, and PsycINFO from inception to January 13th 2016, using terms related to insomnia, cognitive behavioural therapy for insomnia (CBT-I), digital or internet delivery, and randomised trials, with no language restrictions. A meta-analysis of 11 randomised controlled trials of digital CBT-I reported small-to-large effects on insomnia severity, but included both therapist-guided and automated dCBT-I, trials were small and did not retain the control group for follow-up assessments beyond 6 months. Upon analysing these long-term follow-up outcomes, the literature-search was updated on August 20th, 2025, to capture more recent evidence. An updated 2025 meta-analysis of fully automated digital CBT-I, including 29 trials, found moderate-to-large effects but only three trials with follow-up beyond 6 months. These three trials had substantial limitations in population, outcomes, or design. A 2024 meta-analysis identified four trial-based economic evaluations, all ≤6 months and only one assessing the cost-effectiveness of automated dCBT-I. Because programme costs are largely incurred upfront while benefits accrue over time, long-term clinical and economic data for fully automated dCBT-I in general-population settings were lacking.

**Added value of this study**

This is the first trial to report 2-years outcomes for automated digital CBT-I in the general population, with no clinician contact during enrolment, screening, treatment, or follow-up. A self-guided digital assessment followed by automated CBT-I produced sustained improvements in insomnia severity two years after the intervention and was cost-effective compared with digital patient education from societal, healthcare, employer, and patient perspectives. The intervention also increased quality-adjusted life years (QALYs) over 2 years. We also show that successful insomnia treatment is associated with improved health-related outcomes two years later. As the longest randomised controlled evaluation of this low-threshold digital approach, and the first long-horizon trial-based economic evaluation, these findings directly address major gaps in evidence on the durability and cost-effectiveness, of automated dCBT-I.

**Implications of all the available evidence**

The accumulated evidence supports implementing automated digital CBT-I for adults with insomnia in the general population. It is clinically effective and cost-effective across multiple perspectives in a scalable model where participants initiate the intervention without therapist involvement, making it suitable for integration into public or private stepped-care models. Implementation should be accompanied by monitoring of engagement, equity of access, and safety, and supported by pragmatic evaluations to optimise reach and effectiveness in routine practice. Further research is needed to assess its impact in clinical populations and in groups with complex comorbidities, and to determine whether long-term benefits can be enhanced through adaptive delivery models or relapse-prevention strategies.

## Introduction

Insomnia is a chronic condition that affects 12–16% of the general population.^1^ It is characterised by difficulties initiating and/or maintaining sleep, and accompanying impairment in daytime functioning. Long-term adverse outcomes include psychological distress, fatigue, and reduced quality of life. Economic consequences include lower work productivity, higher sick-leave and health service utilisation, medication use, and higher risk for mental and physical illness.^2^^,^^3^ In Norway, annual societal cost of insomnia is around €4.8 billion.^2^ Cognitive behavioural therapy for insomnia (CBT-I) is a recommended first-line treatment for insomnia,^4^ yet access remains limited due to a shortage of trained clinicians and, in many health systems, limited coverage within publicly funded services. To address this, digital CBT-I (dCBT-I) interventions have been developed, ranging from therapist-guided formats to fully automated programmes. Fully automated dCBT-I is interesting, due to its scalability, as it requires no clinical personnel and may substantially reduce costs.

Automated dCBT-I is less clinically effective than individual, face-to-face CBT-I, with large differences post-treatment, and moderate differences in the medium term (6-months).^5^ However, compared with no treatment, waitlist controls, or self-help, automated dCBT-I is more effective in reducing insomnia severity and alleviating depressive symptoms.^6^, ^7^, ^8^ Published randomised controlled trials (RCTs) are of short duration or lack a control group for long-term evaluations. Three trials provide follow-up data beyond 6-months (1–1.5 years) against control groups, but these were limited in scope.^9^, ^10^, ^11^ One focused exclusively on depression prevention,^9^ and another restricted inclusion to individuals with depressive and insomnia symptoms.^11^ The third study reported insomnia and sleep-related outcomes at 1-year follow-up but did not assess broader health-related outcomes.^10^ As such, evidence is lacking for the long-term effectiveness of automated dCBT-I in the general population, with no outcome data beyond one year.

From a public health perspective, we know insomnia is prospectively associated with mental illness.^3^ Further, dCBT-I has shown beneficial effects on depression and fatigue^8^^,^^9^^,^^12^^,^^13^ and acute improvements in insomnia severity during dCBT-I mediate changes in both mental and physical health outcomes.^13^ It is unclear whether insomnia treatment improves other aspects of health or functioning over time, nor whether such benefits are mediated by improvements in insomnia severity.^9^, ^10^, ^11^ Likewise, long-term impacts of dCBT-I on general practitioner (GP) visits or other healthcare utilisation are unknown.

There is limited understanding of the costs and economic implications of screening and treating insomnia across healthcare and employment.^14^ There are few economic evaluations of dCBT-I and methodological weaknesses limit the generalizability of the findings. Most studies have assessed guided dCBT-I, with only two observational studies examining automated interventions.^15^^,^^16^ A 2024 study found dCBT-I cost-effective in stroke survivors, but the small sample and 8-week horizon rendered it underpowered to detect differences in economic outcomes.^15^ Another study found potential reductions in health service costs, but the findings were based on a single-arm retrospective claims analysis.^16^ Regarding societal costs, recent meta-analyses indicate that guided dCBT-I may increase work productivity,^17^^,^^18^ and fully automated dCBT-I may reduce presenteeism through an improvement in insomnia severity.^19^ However, the cost-effectiveness of automated dCBT-I remains largely unexplored and the lack of robust economic evidence is a major barrier to widespread adoption.

To address shortcomings in the knowledgebase, we performed a large-scale RCT of fully automated dCBT-I compared with online patient education about insomnia (PE) across 2-years. The content of PE is based on interventions recommended by insomnia-focused websites and represents a realistic comparator and scalable alternative to face-to-face therapy in a general population setting.^10^^,^^20^ In the pre-published trial protocol, we specified clinical effectiveness at 9-weeks as the primary outcome, with outcomes at 6-months and 2-years as secondary.^20^ Our previous publication demonstrated that dCBT-I is more effective compared with PE (d = 1.21) at 9-weeks.^12^ This report evaluates: 1) between-group differences in insomnia severity at 2-year follow-up and cost-effectiveness of the intervention from a societal perspective with a 2-year time-horizon; and 2) between-group differences on levels of daytime impairment (fatigue, psychological distress, quality of life), sleep diary outcomes, and whether improvement in insomnia severity at post-intervention mediated long-term changes in levels of psychological distress and fatigue, at 2-years.

## Methods

The reporting of the trial follows the CONSORT 2025 guidelines for RCTs.^21^ Cost-effectiveness analyses follow the CHEERS 2022 checklist.^22^ The trial was an investigator-initiated study sponsored by the Norwegian Council of Research, and the Liaison Committee for Education, Research, and Innovation in Central Norway. The patient user group of the Central Norway Health Trust (Regionalt brukerutvalg, Helse Midt-Norge) contributed to the trial by providing input on study aims, assessments, and the online screening process, and approved the final protocol submitted for ethical review.

### Study design

A pragmatic two-arm, parallel-group, superiority randomised controlled trial comparing fully automated dCBT-I with PE in a general-population sample of adults in Norway. The study protocol^18^ was approved by the Regional Committee for Medical and Health Research Ethics in Southeast Norway (2015/134) and the trial was pre-registered with the ClinicalTrials.gov website (NCT02558647). A full trial overview is presented elsewhere^12^^,^^20^ with detailed information on the trial’s rationale, eligibility criteria, randomisation and masking procedures. Here we summarise the key methods.

The trial was not overseen by a Data and Safety Monitoring Board (DSMB), as Norwegian health research regulations at the time of data-collection did not require a DSMB for trials not involving new drugs or medical devices.

### Participants

Adults from the general population in Norway were recruited between February 2016 and July 2018 via advertisements across media outlets and primary care offices, directing interested individuals to a website for further information. Potential participants then self-registered and completed an eligibility screening assessment. Inclusion criteria were: (1) age ≥18 and (2) an Insomnia Severity Index (ISI) score ≥12.^23^ Exclusion criteria were: (1) current shift-work including night-time work hours, (2) risk of sleep apnea or hypersomnia (Epworth Sleepiness Scale score >10^24^ or positive endorsement of screening questions), (3) self-reported contraindicated medical condition (i.e., recent cardiac surgery, psychotic disorder, or epilepsy). Race and ethnicity data were not collected. All participants gave a written informed consent in accordance with the Revised Declaration of Helsinki. Participants received no compensation and were free to withdraw consent any time.

### Randomisation and masking

Eligible participants who provided online written informed consent were randomly assigned (1:1) to dCBT-I or PE via an automated web page. Randomisation was automated and embedded within the study platform, with no access or influence from the research team. Participants were not explicitly informed of their allocation, though the intervention content likely allowed many to infer their assigned group.

### Procedures

Following screening, participants received personal login details via e-mail to access and complete baseline assessments (self-reported demographics, sleep-diaries, pre-specified questionnaires, and information about ongoing medical or mental health conditions).^12^ Eligible participants were then randomised and used their personal login to access the intervention. Email invitations to complete follow-up questionnaires were sent at 9 weeks post-baseline, and again 6-months and 2-years after the 9-week assessment.

The dCBT-I programme, Sleep Healthy Using the Internet (SHUTi),^25^ and the PE website were translated into Norwegian by clinical sleep specialists. SHUTi is an interactive web platform where the components are integrated. By stepwise information, videos, and interactive questions, sleep restriction, stimulus control, cognitive restructuring, sleep hygiene and relapse prevention are conveyed in six “cores”. Each core unlocks 7 days after the previous one, enabling completion in 6 weeks (participants were allowed 9 weeks before post-intervention assessment). Recommendations and feedback are tailored to user data from each core and daily sleep diaries. Participants allocated to PE had immediate access to a static website with information about insomnia (prevalence, causes, etc.) and behavioural recommendations to improve sleep (i.e., stimulus control and sleep hygiene education) that takes 30–45 min to read and a downloadable 7-day sleep diary PE content was adapted from a review of existing insomnia education websites and matched control interventions used in other RCTs of SHUTi.^10^^,^^12^

### Outcomes

We report ISI outcomes at 6-month and 2-year follow-up (the primary outcome findings are reported elsewhere).^12^ The ISI is a validated self-report instrument with strong psychometric properties, widely recommended for use in insomnia research.^23^ It comprises seven items that assess the nature, severity, and functional impact of insomnia symptoms over the past two weeks. A higher score indicates greater severity of insomnia symptoms. The ISI can be used to report response (reduction of ≥8 points on ISI from baseline) and remission (ISI score < 8).

Other outcomes were: The Bergen Insomnia Scale (BIS)^26^ was used as a supplementary insomnia measure since the ISI was used to assess eligibility for participation. The BIS consists of six items, with higher scores indicating greater insomnia severity. At all follow-ups, participants were asked to complete a minimum of 10 days of sleep diaries within a 14-day period using the Consensus Sleep Diary^27^ (which was specifically developed for insomnia research).^23^ Data were extracted on rise time (h), bedtime (h), sleep onset latency (min), wake time after sleep onset (min), early morning awakening (min), total sleep time (h), time in bed (h), and sleep efficiency (%). Psychological distress and fatigue were assessed using the 14-item Hospital Anxiety and Depression Scale (HADS)^28^ and the Chalder Fatigue Questionnaire (CFQ; 11-items measuring physical and psychological fatigue, and the duration and intensity of symptoms).^29^ Higher scores on both scales indicate greater severity. Health-related quality of life (HRQoL) was assessed using the Short Form-6D (SF-6D; derived from the SF-12) with UK general population utility weights applied.^30^ Adverse events were not systematically recorded, but participants were encouraged to contact the study team with any health concerns or technical difficulties. Both PE and CBT-I are considered low-risk interventions. Below we summarize the methods for cost-effectiveness evaluation (see Appendix B for details). Secondary outcomes listed in the trial protocol but not reported here are: Brief Morningness-Eveningness Questionnaire, Brief Dysfunctional Beliefs and Attitudes Scale 16, questionnaire items on pain, physical health, physical activity, and mental health symptoms (Adapted from the Trøndelag Health Study), Alcohol Use Disorders Identification Test- Consumption, electronic media use, internet intervention evaluation, long-term use of sleep-strategies, and negative effects of treatment. These outcomes will be reported in dedicated papers. For a full overview, see list in Appendix C.

### Cost-effectiveness analysis

A within-trial cost–utility analysis estimated the incremental cost-effectiveness of dCBT-I vs. PE over a 2-year horizon. Cost-effectiveness was expressed as an incremental cost-effectiveness ratio (ICER), defined as incremental costs per quality-adjusted life year (QALY) gained. Findings were evaluated against a willingness-to-pay (WTP) threshold of €30,000 per QALY, in line with Norwegian guidance for conditions of low severity.^31^ QALYs were calculated using the area under the utility curve of SF-6D utility scores across the baseline, 9-weeks, 6-month, and 2-year intervals, using the trapezoid rule (Appendix B pp 7). In the base case, we adopted a societal perspective as the intervention was fully automated and offered in a general population setting.

Societal costs included programme, medical, productivity, and patient out-of-pocket expenses. We estimated programme costs for dCBT-I and PE from a lump sum license fee paid to the collaboration partner University of Virginia (Appendix B pp 8). Medical costs included GP visits, outpatient visits and sleep medication use. Health service use was pre-specified to be obtained from registry data^20^ but due to logistical constraints, only patients’ self-reported data at baseline, 6-months and 2-years were used. GP and outpatient visits to psychiatrist or other specialist (yes/no, 3-month recall), and daily sleep medication use (yes/no from sleep diaries), were summarised as odds ratios at 2-years. Volume of visits were estimated for the 2-year follow-up and valued using publicly available unit costs. Days with sleep medication use was converted to costs based on pharmacy maximum retail price for commonly used over-the-counter sleep medicine (Appendix B pp 8–9).

Work productivity and activity impairment were measured using the Work Productivity and Activity Impairment Questionnaire (WPAI)^32^ at the pre-set follow-ups, with responses summarised into hours of absenteeism (1-month recall) and presenteeism (1-week recall), and percentage of activity impairment in daily activities (1-week recall). Productivity costs were estimated from presenteeism and absenteeism, applying the national mean hourly wage in Norway (Appendix B pp 9–10). Costs were valued at 2019 Norwegian kroner and converted to Euros using the exchange rate €1 = 9.85 Norwegian kroner (Appendix B, Supplementary Table S5). No discounting was performed since costs and outcomes, reported at 6-months and 2-years, could not be separated by year.

We supplemented the base case analysis by estimating ICERs from patient, employer, and healthcare perspectives, where in addition to programme costs, we restricted the cost estimates to include out-of-pocket expenses, productivity losses, and medical costs respectively (Appendix B pp 8). We adjusted the time horizon to 6-months to present interim cost-effectiveness results for all perspectives (Appendix B pp 10–11). We performed one-way deterministic analyses to examine how changing programme costs, medical costs, and productivity costs of dCBTI affected the ICER (Appendix B pp 11).

### Statistical analysis

Based on prior trials of dCBT-I vs. PE,^9^^,^^10^ a sample of 486 participants (243 per group) was estimated to provide 80% power (p < 0.05) to detect a moderate-to-large effect on ISI at post-intervention, assuming 50% attrition. To ensure adequate power to detect differences in 2-year follow-up outcomes, including sick leave and health care use, the target sample was increased a priori to 1500 participants (see protocol for details).^18^

To examine the effects of dCBT-I on clinical outcomes, we used a repeated-measures model with categorical time-points (post-intervention, 6-months, and 2-years) in Mplus. The model was parameterised in a wide-data structural equation modelling (SEM) framework as a latent growth model with time-specific change factors to estimate mean change from baseline at each follow-up and between-group differences in these changes. The time-specific change factors had variances fixed to zero (i.e., no random slopes), and the resulting estimates are equivalent to those from a standard repeated-measures linear mixed model estimated with a subject-level random intercept. Robust maximum likelihood estimation was used for all Mplus models, assuming data were missing at random (MAR). Between-group standardized effect sizes (Cohen’s d) were calculated as the between-group difference in model-estimated change from baseline divided by the pooled baseline SD.^12^^,^^33^ As standardized effects can differ meaningfully depending on whether endpoint or change-score standardization is used,^34^ we have also added standardized mean differences at each follow-up as a footnote in Table 1.

**Table 1:** Latent growth model estimates for outcomes related to sleep, daytime function, and mental health.

	Digital CBT-I		Patient education (PE)		Intervention effect		
	n	Mean (SD)	n	Mean (SD)	Estimate (95% CI)	Cohen’s d (95% CI)	p value
Insomnia measure							
Insomnia Severity Index (ISI)							
Baseline	867	19.2 (3.9)	853	19.6 (4.0)	–	–	–
6-months follow-up	421	10.2 (6.3)	387	14.0 (5.6)	−3.4 (−4.2 to −2.7)	−0.83 (−1.01 to −0.65)	<0.001
2-years follow-up	303	10.7 (5.9)	284	13.4 (5.9)	−1.8 (−2.7 to −0.9)	−0.43 (−0.64 to −0.22)	<0.001
Bergen Insomnia Scale (BIS)							
Baseline	867	27.9 (7.4)	853	28.0 (7.6)	–	–	–
6-months follow-up	419	15.1 (10.0)	392	19.7 (9.6)	−4.6 (−5.9 to −3.4)	−0.59 (−0.75 to −0.43)	<0.001
2-years follow-up	315	16.5 (9.6)	269	19.9 (9.8)	−2.6 (−4.1 to −1.2)	−0.33 (−0.51 to −0.15)	<0.001
Sleep diary measures							
Sleep onset latency (min)							
Baseline	868	55.0 (42.8)	853	55.7 (47.3)	–	–	–
6-months follow-up	354	31.3 (30.1)	315	41.8 (32.8)	−8.9 (−13.4 to −4.4)	−0.20 (−0.31 to −0.10)	<0.001
2-years follow-up	287	32.6 (27.8)	248	38.8 (32.0)	−4.9 (−10.0 to 0.2)	−0.11 (−0.23 to 0.01)	0.062
Wake after sleep onset (min)							
Baseline	868	45.5 (40.4)	853	44.6 (37.5)	–	–	–
6-months follow-up	354	27.2 (28.3)	315	33.4 (32.2)	−7.0 (−11.4 to −2.5)	−0.18 (−0.29 to −0.06)	0.002
2-years follow-up	287	26.9 (26.6)	248	35.3 (35.3)	−8.8 (−14.2 to −3.4)	−0.23 (−0.37 to −0.09)	0.002
Early morning awakening (min)							
Baseline	868	41.6 (34.0)	853	43.3 (39.2)	–	–	–
6-months follow-up	354	22.9 (22.2)	315	30.3 (24.5)	−6.4 (−10.3 to −2.4)	−0.17 (−0.29 to −0.06)	0.002
2-years follow-up	287	25.7 (25.1)	248	30.2 (25.4)	−2.5 (−7.1 to 2.2)	−0.07 (−0.19 to 0.06)	0.29
Time in bed (hours)							
Baseline	868	8.3 (1.0)	853	8.2 (1.0)	–	–	–
6-months follow-up	354	7.90 (0.97)	315	8.22 (1.05)	−0.32 (−0.46 to −0.19)	−0.31 (−0.44 to −0.18)	<0.001
2-years follow-up	287	8.04 (0.96)	248	8.25 (0.99)	−0.16 (−0.29 to −0.02)	−0.15 (−0.28 to −0.02)	0.021
Total sleep time (hours)							
Baseline	868	5.9 (1.2)	853	5.9 (1.2)	–	–	–
6-months follow-up	354	6.55 (1.12)	315	6.47 (1.25)	0.1 (−0.1 to 0.2)	0.07 (−0.06 to 0.19)	0.29
2-years follow-up	287	6.63 (1.05)	248	6.52 (1.20)	0.1 (0.0–0.3)	0.10 (−0.03 to 0.23)	0.13
Sleep efficiency (%)							
Baseline	868	71.9 (13.4)	853	71.5 (13.3)	–	–	–
6-months follow-up	354	82.9 (10.9)	315	78.7 (11.4)	3.9 (2.4–5.4)	0.29 (0.18–0.40)	<0.001
2-years follow-up	287	82.5 (10.5)	248	79.0 (11.8)	3.0 (1.3–4.7)	0.22 (0.09–0.35)	0.001
Daytime function and mental health measures							
Psychological distress (HADS total score)							
Baseline	867	13.2 (6.9)	853	13.4 (7.2)	–	–	–
6-months follow-up	396	9.9 (6.9)	363	10.6 (6.8)	−0.6 (−1.3 to 0.1)	−0.08 (−0.18 to 0.01)	0.093
2-years follow-up	310	10.9 (7.1)	267	11.7 (7.2)	0.3 (−0.5 to 1.2)	0.05 (−0.08 to 0.17)	0.464
Chalder Fatigue Questionnaire (CFQ)							
Baseline	867	20.8 (5.9)	853	20.9 (6.0)	–	–	–
6-months follow-up	409	15.7 (6.8)	379	17.4 (6.3)	−1.5 (−2.3 to −0.6)	−0.24 (−0.37 to −0.10)	<0.001
2-years follow-up	313	15.8 (6.8)	271	17.6 (7.1)	−1.0 (−2.0 to −0.1)	−0.17 (−0.33 to −0.01)	0.040

Endpoint effect sizes (Cohen’s d; between-group difference in model-estimated follow-up means divided by the model-implied pooled SD at that follow-up): ISI_6m = −0.69, ISI_2yrs = −0.39; BIS_6m = −0.51, BIS_2yrs = −0.28; SOL_6m = −0.27, SOL_2yrs = −0.15; WASO_6m = −0.20, WASO_2yrs = −0.23; EMA_6m = −0.33, EMA_2yrs = −0.16; TIB_6m = −0.28, TIB_2yrs = −0.11; TST_6m = 0.13, TST_2yrs = 0.16; SE_6m = 0.39, SE_2yrs = 0.29; HAD_6m = −0.11, HAD_2yrs = 0.02; CFQ_6m = −0.25, CFQ_2yrs = −0.18.

Sensitivity analyses were performed to examine the impact of missing data at follow-up under various missing-not-at-random (MNAR) conditions, using pattern-mixture models (PMMs). Participants with complete outcome data at both follow-ups constituted the complete-case group, whereas participants with incomplete data at either time point constituted the incomplete-case group. In the first model (PMM1), separate effect estimates were generated for the complete-case and incomplete-case groups, and model constraints were then applied to derive a weighted average across groups. Because reasons for missingness may differ between treatment conditions, a second model (PMM2) extended PMM1 by stratifying also by treatment group, thereby estimating effects separately in four groups (intervention/complete-case, intervention/incomplete-case, control/complete-case, and control/incomplete-case), followed by the calculation of a weighted average effect estimate across these strata. Finally, we conducted a delta-adjusted tipping-point sensitivity analysis for ISI at 2-years (PMM3), where the primary repeated-measures model was re-estimated under a set of prespecified MNAR additive departures (δ, in ISI points) on the expected outcome among participants with missing ISI. As a conservative scenario, δ was applied to the dCBT-I group only, while the control group followed MAR. We evaluated a grid of δ values and identified the tipping point at which the 2-year between-group difference in change from baseline was no longer statistically significant.

Group differences in treatment response and remission were evaluated by comparing the proportions of participants meeting ISI-based criteria using Pearson’s chi-square test and the Newcombe hybrid score confidence interval for the difference in proportions between dCBT-I and PE.

Post-hoc mediation analyses tested whether dCBT-I effects on long-term psychological distress and fatigue were mediated by post-treatment insomnia severity. Analyses used Stata’s *mediate* command, which applies causal mediation analysis in the counterfactual framework, decomposing the total effect into an average direct effect and an average causal mediation effect. Models were adjusted for age and sex, and confidence intervals were derived from 1000 bootstrap replications.

In the cost-effectiveness analyses, linear mixed models compared mean costs, SF-6D values, and QALYs between groups in the base case, with fixed effects specified for group, time, and their interaction, and a random intercept for individuals. No baseline adjustment was performed. Non-parametric bootstrapping was used to quantify uncertainty surrounding the ICER-estimate by resampling 1000 times from incremental costs and incremental QALYs. The resulting paired incremental cost and QALY replicates were displayed on the cost-effectiveness plane and used to construct cost-effectiveness acceptability curves across a range of WTP thresholds. To account for uncertainty surrounding missing data, chained equations and predictive mean matching (with *k* nearest neighbour = 5) were applied for multiple imputation regressing on covariates (ISI, BIS, HADS and fatigue scores; age, sleep medicine use, sex, health service use, SF-6D index and costs) stratified by group. Imputation was done for societal and health sector perspective cost categories and utility values for all timepoints in the two trial groups. A total of 50 imputed samples were generated, which were subsequently combined using Rubin's rule. Finally, we explored data MNAR scenarios by conservatively assuming the intervention group experienced 5%–20% lower QALYs and 5%–20% higher costs than observed (Appendix B pp 14).

All reported analyses were conducted on the intention-to-treat population. Statistical analyses were conducted in Mplus version 8.2, STATA version 18.5, and R version 4.4.2. Statistical significance was defined as two-sided α < 0.05.

### Role of the funding source

The study funder was not involved in the design, data collection, analysis, interpretation, or manuscript preparation.

## Results

### Clinical effectiveness analyses

Participants were recruited between Feb 26, 2016, and July 1, 2018. A total of 1720 participants were included in the intention-to-treat population, with a mean age of 44.4 years (SD 13.9; range 18–90). Of these, 1167 (67.8%) were female, and 1074 (62.4%) were married or cohabiting. The sample had a mean of 16.3 years (SD 2.9) of education. Comorbidity was common: 589 participants (34.3%) reported a mental health condition, 211 (12.3%) reported a medical condition, and 199 (11.6%) reported both mental and medical comorbidities. About 40% reported having experienced sleep problems for more than 10 years (see Appendix A, Supplementary Table S1 for further baseline characteristics).

**Fig. 1:**
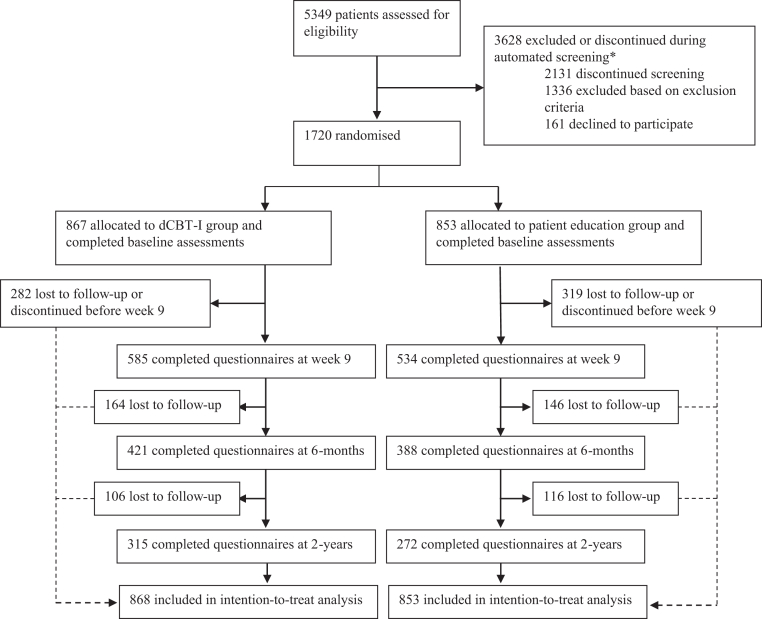
Trial profile.

At 6-months follow-up, 421 participants (49%) in the dCBT-I group and 388 (45%) in the PE group completed the main clinical outcome measure (ISI) (Fig. 1). At 2-years follow-up, 315 (36%) in the dCBT-I group and 272 (32%) in the PE group provided ISI data, with a median follow-up duration of 28.3 months (IQR 22.6 to 34.0) from baseline. In the dCBT-I group, 402 (46%) completed all six treatment cores during the intervention period (see Vedaa et al., 2020 for details).^12^

As shown in Table 1, we observed significant between-group differences for insomnia severity in favour of dCBT-I at all timepoints, with an estimated mean difference of −1.77 (95% CI: −2.65 to −0.90; p < 0.001; Cohen’s *d* = −0.43) at 2-year follow-up (with similar patterns for the BIS). Rates of response and remission were consistently higher in dCBT-I (Appendix A, Supplementary Table S2). Sensitivity analyses showed similar ES for the ISI outcome at the 6-month follow-up (PMM1: d = −0.87 [95% CI: −1.08 to −0.66]; PMM2: d = −0.86 [95% CI: −1.07 to −0.66]). At 2-yeas follow-up, the estimated ES under the MNAR assumption were slightly lower than those reported in Table 1 (PMM1: d = −0.28 [95% CI −0.54 to −0.02]; PMM2: d = −0.30 [95% CI: −0.55 to −0.04]). In a delta-adjusted MNAR tipping-point analysis, statistical significance of the between-group difference was lost when missing 2-year outcomes in the dCBT-I group were assumed to be 1.36 ISI points worse than predicted under MAR, while the between-group difference was attenuated to −0.88 ISI points.

Further positive effects were observed on some, but not all, other clinical outcome measures at the 2-year follow-up (Table 1). Sleep diary data confirmed greater improvements in the dCBT-I group in several sleep parameters at the 2-year follow-up, including WASO, TIB, and SE.

### Mediation analyses

The mediation analyses are shown in Appendix A, Supplementary Table S3. dCBT-I had a significant indirect effect on HADS at 2-years through ISI at 9-weeks. The effect of dCBT-I on fatigue symptoms at 2-years was fully mediated through ISI at 9-weeks.

### Cost-effectiveness analyses

There were no long-term effects on HRQoL (SF-6D index). The odds of sleep medication use were 52% lower in the dCBT-I group at 6-months. By 2-years, the difference in medication use was not statistically significant (Table 2).

**Table 2:** Linear mixed model estimates on outcomes related to cost-effectiveness.

	Digital CBT-I		Patient Education (PE)		Intervention effect	
	n	Mean (SD)	n	Mean (SD)	Estimate (95% CI)	p value
SF-6D index						
Baseline	868	0.587 (0.077)	853	0.587 (0.078)		
6-months follow-up	408	0.623 (0.088)	377	0.612 (0.076)	0.010 (−0.001 to 0.021)	0.068
2-years follow-up	312	0.621 (0.077)	270	0.614 (0.084)	0.002 (−0.010 to 0.014)	0.72
Visited a GP in the last 3 months						
Baseline («Yes»)	867	607 (70.0%)	851	576 (67.7%)		
6-months follow-up («Yes»)	397	221 (55.7%)	363	217 (59.8%)	0.692 (0.457–1.046)	0.08
2-years follow-up («Yes»)	310	165 (53.2%)	268	172 (64.2%)	0.502 (0.316–0.797)	0.004
Visited a psychiatrist in the last 3 months						
Baseline («Yes»)	867	61 (7.0%)	852	75 (8.8%)		
6-months follow-up («Yes»)	397	29 (7.3%)	363	22 (6.1%)	1.73 (0.74–4.04)	0.206
2-years follow-up («Yes»)	310	20 (6.5%)	268	14 (5.2%)	1.90 (0.71–5.12)	0.202
Visited a specialist in the last 3 months						
Baseline («Yes»)	867	150 (17.3%)	852	135 (15.8%)		
6-months follow-up («Yes»)	397	69 (17.4%)	363	66 (18.2%)	0.78 (0.45–1.34)	0.37
2-years follow-up («Yes»)	308	44 (14.3%)	268	48 (17.9%)	0.60 (0.32–1.11)	0.10
Absenteeism (Hrs/week)						
Baseline	578	2.43 (4.3)	580	2.09 (3.5)		
6-months follow-up	267	1.66 (3.8)	255	1.65 (3.5)	−0.35 (−1.04 to 0.34)	0.32
2-years follow-up	210	0.79 (2.3)	178	0.91 (2.5)	−0.42 (−1.19 to 0.36)	0.29
Presenteeism (Hrs/week)						
Baseline	583	11.03 (10.3)	588	11.12 (9.7)		
6-months follow-up	269	7.09 (9.7)	261	8.70 (9.7)	−1.41 (−3.14 to 0.31)	0.11
2-years follow-up	212	6.69 (9.0)	181	8.00 (9.9)	−0.71 (−2.66 to 1.25)	0.48
Activity impairment (%)						
Baseline	867	41.6 (29.6)	853	43.9 (28.8)		
6-months follow-up	400	27.8 (28.7)	365	33.0 (29.5)	−2.92 (−6.98 to 1.14)	0.16
2-years follow-up	311	28.0 (28.9)	268	31.7 (30.3)	0.69 (−3.88 to 5.25)	0.77
Sleep medication use						
Baseline («Yes»)	868	495 (57%)	853	519 (60.8%)		
6-months follow-up («Yes»)	354	142 (40.1%)	315	166 (52.7%)	0.4842 (0.28–0.84)	0.010
2-years follow-up («Yes»)	287	128 (44.6%)	248	124 (50.0%)	0.8557 (0.47–1.55)	0.61

**Fig. 2:**
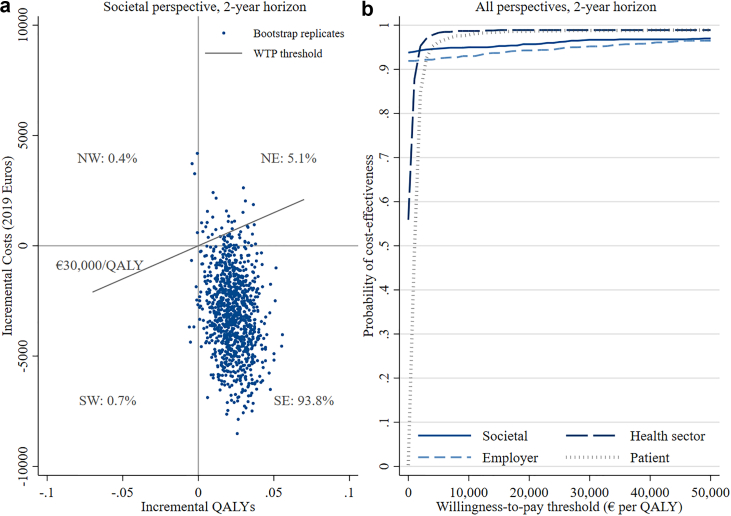
Cost-effectiveness of dCBT-I compared with online patient education at 2 years. Note: a) Cost-effectiveness plane representing bootstrapped mean differences in costs and QALYs for dCBT-I compared with online patient education in the societal perspective at 2 years and b) Cost-effectiveness acceptability curves showing the probability of dCBT-I being cost-effective compared to online patient education at different willingness-to-pay thresholds per QALY gained in societal, health sector, employer and patient perspectives at 2 years.

Over the 2-year horizon the linear mixed models estimated that the dCBT-I group had an incremental gain of 0.025 QALYs (95% CI: 0.010 to 0.041; p = 0.001) compared with PE (Table 3). The programme costs were €70.16 per patient for dCBT-I and €23.80 per patient for PE. From a societal perspective, dCBT-I generated an incremental saving of -€278 (95% CI: -€1413 to €858) compared with PE. At 2-years, dCBT-I was the dominant strategy with lower costs and QALY gain compared with PE resulting in an ICER of −€10,973 per QALY gained. Bootstrapping the estimates showed a 94% likelihood that the intervention yields lower costs and higher QALYs, and there was a 97% probability that the intervention is cost-effective compared with the WTP threshold of €30,000 per QALY. The cost-effectiveness plane in Fig. 2 displays the uncertainty surrounding the mean ICER, whereas the accompanying acceptability curves display the probability of cost-effectiveness in all perspectives relative to a range of WTP thresholds.

**Table 3:** Estimates of costs, QALYs, and the incremental treatments costs.

	dCBT-I		Patient Education (PE)		Incremental	
	n^a^	Mean (95% CI)^b^	n^a^	Mean (95% CI)^b^	Mean (95% CI)^b^	p value^b^
Intervention costs (€)						
Programme costs	868	70.16	853	23.80	46.36	
Direct medical costs (€)						
GP	310	224 (215–234)	268	271 (261–281)	−47 (−61 to −33)	<0.001
Psychiatrist outpatient	310	19 (16–22)	268	15 (11–18)	4 (−1 to 9)	0.11
Other specialist outpatient	308	45 (39–50)	268	53 (47–58)	−8 (−16 to −1)	0.040
Sleep Medication	263	41 (38–45)	224	53 (50–57)	−12 (−16 to −7)	<0.001
Sum of medical costs	310	323 (310–336)	268	383 (369–397)	−60 (−80 to −41)	<0.001
Productivity costs (€)^c^						
Absenteeism	210	2794 (2327–3261)	178	3251 (2744–3758)	−457 (−1147 to 232)	0.18
Presenteeism	212	21,847 (20,076–23,617)	181	26,519 (24,604–28,434)	−4673 (−7281 to −2065)	<0.001
Sum of productivity costs	212	24,613 (22,581–26,645)	182	29,547 (27,355–31,739)	−4934 (−7923 to −1945)	0.001
Total costs (€)	868	6183 (5383–6983)	853	6461 (5654–7267)	−278 (−1413 to 858)	0.66
QALYs	312	1.336 (1.325–1.346)	270	1.310 (1.299–1.322)	0.025 (0.010–0.041)	0.001

a *n* reporting at 2 years follow-up.

b Estimates from linear mixed models.

c Estimated for those in full or part time employment.

Extending the base case analyses to varying perspectives and time horizons showed that dCBT-I is cost-effective in the health care sector, employer and patient perspectives at 2-years compared with PE (Appendix B, Supplementary Table S9). It was also cost-effective in all perspectives at 6-months but the incremental QALYs (d = 0.010; 95% CI: 0.003 to 0.015; p = 0.002) were lower when compared with the 2-year time horizon (Appendix B, Supplementary Table S10). One-way deterministic analyses indicated that, over the 2-year horizon, compared with PE, dCBT-I remained cost-effective until a maximum programme cost of €1110 per patient in the societal perspective and €789 in the healthcare sector perspective, after which it exceeded the WTP threshold. The ICER, relative to the WTP threshold, was robust to one-way increases in the dCBT-I group’s base case medical costs (up to 50%), and productivity costs (up to 10%) (Appendix B, Supplementary Table S11).

After imputing missing values, the estimated incremental QALYs were 0.014 (95% CI: −0.002 to 0.030) and incremental costs were −€1750 (95% CI: −€5446 to €1946), yielding an ICER of −€125,833 per QALY gained in favour of dCBT-I compared with PE (Appendix B, Supplementary Table S13). In MNAR scenario analyses, dCBT-I remained cost-effective when costs in the dCBT-I group only were increased by up to 20% in the health sector perspective and up to 10% in the societal perspective. Reducing imputed QALYs by 5% in the dCBT-I group resulted in lower effectiveness compared with PE (Appendix B, Supplementary Table S14).

## Discussion

This trial demonstrates the clinical effectiveness of fully automated dCBT-I, compared with a digital PE control, over two years, the longest controlled follow-up of dCBT-I to date. Participants were self-referred and underwent automated eligibility screening before randomisation, reflecting a scenario that closely resembles how such interventions could be disseminated at scale in a stepped-care model. Within this context, dCBT-I produces long-term sustained reductions in insomnia severity, and is likely cost-effective from societal, healthcare, employer, and patient perspectives compared with PE. These findings address important gaps in the evidence-base regarding longer-term effectiveness, cost-effectiveness, and the viability of fully automated digital interventions for insomnia. Combined with evidence from shorter-term trials,^6^ these findings may help inform clinical implementation and health-service planning for digital sleep medicine in the general population. However, the findings require replication and our conclusions are limited by missingness in the 2-year outcome data.

Consistent with a previous long-term uncontrolled study of dCBT-I in the general population,^35^ we found that within-group ISI score reductions were sustained for dCBT-I. However, between-group differences relative to PE diminished over two years, consistent with shorter-term RCTs of digital^6^ and other CBT-I delivery formats.^36^ Such attenuation of between-group effects over extended follow-up (ES 0.4) is expected and may reflect sustained improvements in the intervention group alongside gradual symptom improvement in comparator groups. While a Cohen’s *d* of about 0.5 is suggested as a benchmark for clinically meaningful short-to medium-term differences on the ISI,^5^^,^^37^ smaller effects may still be considered clinically meaningful in a long-term context. Also, effects of this magnitude can translate into meaningful population-level benefits when delivered at scale. We observed a similar trend for sleep medication use, with reductions relative to PE evident over the first six months. Taken together, these findings indicate that dCBT-I accelerates insomnia improvements, while its relative advantage against comparators narrows over time.

Other clinical outcomes showed a mixed pattern. Fatigue remained significantly lower in the dCBT-I group throughout follow-up, whereas psychological distress did not differ between groups. However, post-intervention changes in insomnia severity emerged as a key mediator of longer-term outcomes in both fatigue and psychological distress. These findings support a mechanistic model in which insomnia acts as a modifiable pathway influencing broader psychological wellbeing long-term, even in the absence of direct intervention effects on mood. Among sleep parameters, wake after sleep onset was reduced over time in the dCBT-I group, although no differences were observed in total sleep time. Consistent with short-term trials, these findings underscore that dCBT-I can produce lasting improvements in sleep, primarily by enhancing sleep continuity and subjective sleep perception over time.^38^

Effects on healthcare utilisation emerged later. Relative to PE, GP visits in the dCBT-I group were significantly reduced only at two years, suggesting that downstream benefits may take longer to manifest. These findings indicate that low-threshold implementation of dCBT-I in the general population could improve primary care efficiency, where e.g., 8% of GP visits in Norway result in sleep medication prescriptions.^39^ Although the study was powered to detect differences in healthcare use,^20^ reported findings are based on self-report data collected at three time points. Using subjective recall and issues with missing data may limit the scope and precision of health service cost estimates and replication using objective healthcare data is warranted.

Exploring the cost-effectiveness of dCBT-I in alternative perspectives is relevant because the intervention is fully automated and can be implemented outside the health care system. While our societal perspective excluded important cost categories, such as inpatient stays, allied health services and informal care, in line with a recent meta-analysis,^17^ lower productivity losses, especially presenteeism, were a major driver of the cost-effectiveness in societal and employer perspectives. In contrast to the societal perspective, within the healthcare sector and patient perspectives, where productivity costs were excluded, cost-effectiveness was achieved through estimated reductions in GP visits and sleep medication use. Notably, while the base case incremental QALYs were small but statistically significant, reducing the time horizon from 2-years to 6-months reduced the incremental QALYs. These results are consistent with previous studies suggesting that dCBT-I can reduce productivity losses and healthcare costs, but its impact on QALYs is modest.^17^ They also indicate that longer follow-up periods are necessary to fully capture the economic benefits of the intervention. We interpret our cost-effectiveness estimates with caution however, since the lack of discounting may have limited their precision, whereas the choice of PE as a comparator may have limited the generalizability of our findings. Additionally, the market price of dCBT-I–subject to scale, pricing model and payer^40^–may vary from our estimated programme costs. We addressed this price uncertainty through one-way deterministic analyses. While our estimated maximum programme costs per patient compare favourably to similar interventions,^17^ they are independent of demand elasticity.

A limitation of the study is increasing attrition across follow-up assessments. Response rates at 6-months were comparable or higher than previous trials of similar design and shorter follow-up,^10^^,^^13^^,^^41^ but the low response rate at 2-years may have introduced bias in estimates of clinical and cost-effectiveness. Missing data are an inevitable challenge in long-term digital intervention trials, especially automated studies, and sensitivity to missingness assumptions is critical for robust inference. MNAR sensitivity of ISI estimates were like the primary analysis at 6-months, but attenuated at 2-years. In a conservative delta-adjusted MNAR tipping-point analysis, statistical significance at 2-years was not maintained following a modest deviation from MAR (δ = 1.36 ISI points) in the dCBT-I group; indicating that conclusions regarding durability should be interpreted with caution. Similarly, the incremental costs remained robust to multiple imputation and various MNAR scenarios, but incremental QALYs reduced after imputation.

Engagement with the fully automated programme was variable, with only 46% of dCBT-I participants completing all six cores. In our primary post-treatment report, a complier-average causal effect (CACE) analysis suggested larger improvements among participants who completed the full programme.^12^ This suggests the degree of treatment completion, and thus the effective dose of dCBT-I received is a driver of outcome. Moreover, the behavioural adherence, or the extent to which patients apply treatment recommendations is also relevant for outcome in CBT-I.^42^ This points to a central tension in the implementation of fully automated dCBT-I: maximising scalability and reach while ensuring sufficient individual engagement and adherence to achieve meaningful benefit. One way to address this is via a stepped-care model. By offering fully automated dCBT-I as a first step, this circumvents the inherent scalability and availability issues of face-to-face CBT-I and aligns with recommendations that CBT-I be offered as first-line treatment.^4^ Although more costly than providing PE as the first-line intervention, this study indicates that programme costs are offset by savings in healthcare use and productivity gains long-term. Ideally, baseline complexity along with continuous monitoring of symptoms, adherence, and early response could identify individuals unlikely to benefit sufficiently from self-guided delivery and drive timely escalation to therapist-assisted digital or face-to-face CBT-I.^43^ More research is needed to understand the precursors to non-response in fully automated dCBT-I, alongside continuing enhancements to the user experience of these programs.

Taken together, our findings suggest that fully automated dCBT-I, delivered with automated eligibility screening and without therapist involvement, is associated with improvements in insomnia severity that persist over two years and appeared to be cost-effective compared with PE. However, the magnitude of benefit attenuates over time, and long-term inferences are sensitive to missing-data assumptions. The findings offer support for the potential of dCBT-I as a scalable first-line intervention for insomnia in routine care, while underscoring the need for further research on implementation and long-term optimisation.

## Contributors

HK and BS acquired funding. HK, ØV, and BS conceptualised the study. ØV oversaw the data-collection. CLV, ZAK, ORFS, and DV curated data for analysis. Formal analyses were performed by ORFS, ZAK, and DV. Visualization was done by CLV, ZAK, and DV. HK supervised the study and provided mentorship together with VH and JS. HK, CLV, ZAK, and DV wrote the original draft. Review and editing were done by all coauthors. ORFS, ØV, CLV, ZAK, HK, and DV has directly accessed and verified the data. The corresponding author held the final responsibility over the decision to submit the manuscript for publication.

## Data sharing statement

Norwegian regulation does not allow for the sharing of these data after completion of the study. Statistical code is available upon reasonable request from the corresponding author.

## Declaration of generative AI and AI-assisted technologies in the writing process

During the preparation of this work the authors used ChatGPT (GPT 4o and GPT-5) to assist with language and flow. After using this tool/service, the authors reviewed and edited the content as needed and take full responsibility for the content of the publication. ChatGPT was not used to generate scientific content, conduct analyses, or interpret data.

## Declaration of interests

LMR reports having equity ownership in BeHealth Solutions, LLC, who originally licensed the Sleep Healthy Using the Internet (SHUTi) program from the University of Virginia. Somryst, a commercial Prescription Digital Therapeutic for insomnia, was developed based on the SHUTi program by Pear Therapeutics who subsequently sold their license to Nox Health. Nox Health has a royalty agreement with BeHealth Solutions, LLC and the UVA Licensing and Venture Group. Dr. Ritterband is a consultant of Nox Health. These companies had no role in preparing this manuscript. The terms of this arrangement have been reviewed and approved by the University of Virginia in accord with its conflict of interest policy.

## Appendix A Supplementary data

Protocol
