# Supplementary material for: Long-term clinical and cost-effectiveness of a fully automated digital cognitive behavioural therapy for insomnia: 2-year follow-up of a single-blind, superiority, randomised controlled trial
Source: Lancet Reg Health Eur. 2026 May 8;66:101691. doi: 10.1016/j.lanepe.2026.101691 (PMC13186012; doi:10.1016/j.lanepe.2026.101691)
Supplement: Appendix A–C [file mmc2.docx]

**Supplementary Materials for:**Long-term clinical and cost-effectiveness of a fully automated digital cognitive behavioural therapy for insomnia: 2-year follow-up of a single-blind, superiority, randomised controlled trial

**Table of Contents**

[Appendix A: Baseline, responder and mediation tables 2](#_Toc223014943)

[Table S1. Baseline characteristics 3](#_Toc223014944)

[Table S2. Responders and remitters in dCBT-I and PE over all follow-up timepoints. 4](#_Toc223014945)

[Table S3. Mediation analysis of the effect of dCBT-I through ISI on HADS and CFQ 5](#_Toc223014946)

[Appendix B: Heath Economic Analysis 7](#_Toc223014947)

[1. Calculating Quality Adjusted Life Years (QALYs) 7](#_Toc223014948)

[2. Estimating Costs 7](#_Toc223014949)

[2.1 Valuing costs 7](#_Toc223014950)

[Table S5. Unit costs in 2019 Euros 7](#_Toc223014951)

[2.2 Calculation of costs 8](#_Toc223014952)

[Table S6. Costs in various perspectives 8](#_Toc223014953)

[Table S7. Illustrative example of estimating cost by category and by perspective 8](#_Toc223014954)

[Programme costs 8](#_Toc223014955)

[Medical Costs 8](#_Toc223014956)

[Out of pocket costs 9](#_Toc223014957)

[Table S8. Patients’ out-of-pocket medical costs at 2 years. 9](#_Toc223014958)

[Productivity Costs 9](#_Toc223014959)

[3. Sensitivity Analyses 10](#_Toc223014960)

[3.1 Sensitivity Analysis 1: Varying Perspectives 10](#_Toc223014961)

[Table S9. Incremental costs and QALYs in societal, healthcare sector, employer and patient perspectives at 2-years 10](#_Toc223014962)

[3.2 Sensitivity Analysis 2: Varying Time Horizon 10](#_Toc223014963)

[Table S10. Incremental costs and QALYs in societal, healthcare sector, employer and patient perspectives at 6-months 11](#_Toc223014964)

[3.3 Sensitivity Analysis 3: Varying costs – One-way deterministic 11](#_Toc223014965)

[Table S11. Change in incremental cost-effectiveness ratio (ICER) relative to change in programme costs, medical costs and productivity costs of dCBT-I 11](#_Toc223014966)

[3.4 Sensitivity Analysis 4: Imputing missing data 11](#_Toc223014967)

[Table S12. Association of selected demographic characteristics and symptom scores with SF-6D missing data at 2 years 12](#_Toc223014968)

[Table S13. Multiply imputed costs and QALYs in societal and healthcare sector perspectives at 2-years 13](#_Toc223014969)

[3.5 Sensitivity Analysis 5: Imputing with missing not at random scenarios 14](#_Toc223014970)

[Table S14. Incremental costs and QALYs in societal and health care sector perspectives at 2-years in missing not at random scenarios 14](#_Toc223014971)

[Appendix C: Secondary outcomes listed in the trial protocol that are not reported here 15](#_Toc223014972)

[References 16](#_Toc223014973)

#

# **Appendix A: Baseline, responder and mediation tables**

| **Table S1. Baseline characteristics** | | | |
| --- | --- | --- | --- |
| Characteristics | dCBT-I (n=867) | PE (n=853) | Total (n=1720) |
| Age, mean (SD), years | 44·2 (14·1) | 44·7 (13·8) | 44·4 (13·9) |
| Female, No. (%) | 596 (68·7) | 571 (66·9) | 1167 (67·8) |
| Education, mean (SD), years | 16·4 (3·0) | 16·2 (2·9) | 16·3 (2·9) |
| Employment status |  |  |  |
| Full-time employment, No. (%) | 427 (49·2) | 427 (50·1) | 854 (49·6) |
| Part-time employment, No. (%) | 122 (14·1) | 120 (14·1) | 242 (14·1) |
| Unemployed, No. (%) | 61 (7·0) | 46 (5·4) | 107 (9·2) |
| Retired, No. (%) | 74 (8·5) | 74 (8·7) | 148 (8·6) |
| Student, No. (%) | 69 (7·9) | 67 (7·9) | 136 (7·9) |
| Other, No. (%) | 114 (13·1) | 119 (14·0) | 233 (13·5) |
| Married/Cohabiting with partner, No. (%) | 539 (62·2) | 535 (62·7) | 1074 (62·4) |
| Children living in the household, No. (%) | 303 (34·9) | 317 (37·2) | 620 (36·1) |
| Sleep problem duration |  |  |  |
| Less than 6 months, No. (%) | 29 (3·3) | 19 (2·2) | 48 (2·8) |
| 6-11 months, No. (%) | 16 (1·8) | 17 (2·0) | 33 (1·9) |
| 1-5 years, No. (%) | 246 (28·3) | 251 (29·4) | 497 (28·9) |
| 6-10 years, No. (%) | 226 (26·0) | 216 (25·3) | 442 (25·7) |
| >10 years, No. (%) | 343 (39·5) | 338 (39·6) | 681 (39·6) |
| Check email daily, No. (%) | 801 (92·4) | 781 (91·6) | 1582 (92·0) |
| Use internet daily, No. (%) | 851 (98·2) | 837 (98·2) | 1688 (98·2) |
| Very comfortable with internet, No. (%) | 724 (83·5) | 708 (83·0) | 1432 (83·3) |
| Comorbidity |  |  |  |
| Medical condition, No. (%) | 102 (11·8) | 109 (12·8) | 211 (12·3) |
| Mental health condition, No. (%) | 303 (35·0) | 286 (33·6) | 589 (34·3) |
| Medical and mental health condition, No. (%) | 81 (9·4) | 118 (19·8) | 199 (11·6) |
| No comorbidity, No. (%) | 380 (43·9) | 339 (39·8) | 719 (41·9) |
|  |  |  |  |

##### **Table S2. Responders and remitters in dCBT-I and PE over all follow-up timepoints.**

|  | dCBT-I | | |  | PE | | |  | Intervention effect | |
| --- | --- | --- | --- | --- | --- | --- | --- | --- | --- | --- |
|  | N | n | % |  | N | n | % |  | Difference, % (95% CI) | p-value |
| Remitters |  |  |  |  |  |  |  |  |  |  |
| 6-months | 421 | 161 | 38·2 |  | 388 | 53 | 13·7 |  | -24·6 (-30·3 to -18·8) | <·001 |
| 2-years | 315 | 101 | 32·1 |  | 272 | 50 | 18·4 |  | -13·7 (-20·5 to -6·7) | <·001 |
|  |  |  |  |  |  |  |  |  |  |  |
| Responders |  |  |  |  |  |  |  |  |  |  |
| 6-months | 421 | 252 | 59·9 |  | 388 | 111 | 28·6 |  | -31·2 (-37·6 to -24·6) | <·001 |
| 2-years | 315 | 163 | 51·7 |  | 272 | 106 | 39·0 |  | -12·8 (-20·7 to -4·7) | 0·003 |

##### **Table S3. Mediation analysis of the effect of dCBT-I through ISI on HADS and CFQ**

|  |  | Total effect |  |  | Direct effect |  |  | Indirect effect |  |
| --- | --- | --- | --- | --- | --- | --- | --- | --- | --- |
| Assessment | Mediation tested | Estimate (95% CI) | p-value |  | Estimate (95% CI) | p-value |  | Estimate (95% CI) | p-value |
| HADS |  |  |  |  |  |  |  |  |  |
| 6-months | ISI week 9 | -0·64 (-1·66 to 0·38) | 0·217 |  | 0·86 (-0·33 to 2·05) | 0·155 |  | -1·50 (-2·16 to -0·84) | <·001 |
| 2-years | ISI week 9 | -0·63 (-1·99 to 0·74) | 0·367 |  | 1·58 (-·082 to 3·25) | 0·062 |  | -2·21 (-3·20 to -1·23) | <·001 |
| CFQ |  |  |  |  |  |  |  |  |  |
| 6-months | ISI week 9 | -1·64 (-2·60 to -0·68) | 0·001 |  | 0·59 (-0·52 to 1·69) | 0·299 |  | -2·23 (-2·90 to -1·55) | <·001 |
| 2-years | ISI week 9 | -1·70 (-3·02 to -0·39 | 0·011 |  | 0·46 (-1·19 to 2·11) | 0·586 |  | -2·16 (-3·17 to -1·16) | <·001 |

Models were adjusted for age and sex.

**Table S4. Association of selected demographic characteristics and symptom scores with missing data on the ISI at 2 years**

|  |  | **dCBT-I** | | | |  | | **PE** | | | |  |  | |
| --- | --- | --- | --- | --- | --- | --- | --- | --- | --- | --- | --- | --- | --- | --- |
|  |  | Completer | | Non-completer | |  | | Completer | | Non-completer | |  |  |  |
|  |  | Mean /n | sd /% | Mean /n | sd /% | |  | Mean /n | sd /% | Mean /n | sd /% |  | P-value | |
| Baseline N | | 868 | | | | |  | 853 | | | |  |  |  |
| Completed 2 years assessment | | 303 | 35% | 565 | 65% | |  | 284 | 33% | 569 | 67% |  |  |  |
|  |  |  |  |  |  | |  |  |  |  |  |  |  |  |
| Demographic characteristics | |  |  |  |  | |  |  |  |  |  |  |  |  |
|  | Age | 43.20 | 14.12 | 44.73 | 13.94 | |  | 42.26 | 13.51 | 45.84 | 13.83 |  | 0.145 | ^a^ |
|  | Female | 208 | 69% | 441 | 78% | |  | 205 | 72% | 398 | 70% |  | 0.009 | ^b^ |
|  | Married/Cohabiting | 198 | 65% | 341 | 60% | |  | 178 | 63% | 357 | 63% |  | 0.314 | ^b^ |
|  | Years of education | 16.55 | 2.88 | 16.29 | 3.00 | |  | 16.29 | 2.72 | 16.21 | 2.98 |  | 0.533 | ^a^ |
|  | Children living in the household | 120 | 40% | 183 | 32% | |  | 109 | 38% | 208 | 37% |  | 0.262 | ^b^ |
|  | Mobile use (hours per day) | 2.45 | 2.47 | 2.52 | 2.57 | |  | 2.69 | 2.61 | 2.46 | 2.61 |  | 0.250 | ^a^ |
|  |  |  |  |  |  | |  |  |  |  |  |  |  |  |
| BIS | |  |  |  |  | |  |  |  |  |  |  |  |  |
|  | Baseline | 27.04 | 7.48 | 28.34 | 7.33 | |  | 28.02 | 7.36 | 27.97 | 7.73 |  | 0.077 | ^a^ |
|  | 9 weeks | 13.26 | 8.97 | 15.78 | 10.17 | |  | 22.26 | 9.38 | 21.26 | 9.35 |  | 0.003 | ^a^ |
|  | 6 months | 15.16 | 9.15 | 15.11 | 10.68 | |  | 21.12 | 9.59 | 18.94 | 9.55 |  | 0.133 | ^a^ |
|  |  |  |  |  |  | |  |  |  |  |  |  |  |  |
| Fatigue | |  |  |  |  | |  |  |  |  |  |  |  |  |
|  | Baseline | 20.17 | 6.12 | 21.14 | 5.83 | |  | 21.30 | 6.06 | 20.77 | 5.98 |  | 0.014 | ^a^ |
|  | 9 weeks | 14.35 | 6.56 | 16.16 | 7.58 | |  | 17.72 | 6.55 | 17.60 | 6.28 |  | 0.023 | ^a^ |
|  | 6 months | 15.57 | 6.54 | 15.80 | 7.07 | |  | 17.41 | 6.38 | 17.35 | 6.23 |  | 0.759 | ^a^ |
|  |  |  |  |  |  | |  |  |  |  |  |  |  |  |
| HADS | |  |  |  |  | |  |  |  |  |  |  |  |  |
|  | Baseline | 12.97 | 6.58 | 13.37 | 7.08 | |  | 14.29 | 6.79 | 12.97 | 7.33 |  | 0.016 | ^a^ |
|  | 9 weeks | 9.95 | 6.68 | 10.44 | 7.16 | |  | 11.83 | 7.00 | 11.24 | 6.94 |  | 0.217 | ^a^ |
|  | 6 months | 9.93 | 6.90 | 9.94 | 6.94 | |  | 10.95 | 6.73 | 10.46 | 6.88 |  | 0.630 | ^a^ |
|  |  |  |  |  |  | |  |  |  |  |  |  |  |  |
| SF-6D | |  |  |  |  | |  |  |  |  |  |  |  |  |
|  | Baseline | 0.59 | 0.08 | 0.58 | 0.08 | |  | 0.58 | 0.08 | 0.59 | 0.08 |  | 0.013 | ^a^ |
|  | 9 weeks | 0.63 | 0.09 | 0.62 | 0.08 | |  | 0.60 | 0.08 | 0.61 | 0.08 |  | 0.135 | ^a^ |
|  | 6 months | 0.62 | 0.08 | 0.62 | 0.09 | |  | 0.62 | 0.08 | 0.61 | 0.07 |  | 0.522 | ^a^ |

^a^ Generalized linear model; ^b^ Logistic regression. P-values represent a test of the interaction between missingness on ISI and group on the listed outcome variables.

# **Appendix B: Heath Economic Analysis**

## 1. Calculating Quality Adjusted Life Years (QALYs)

Health-related quality of life (HRQoL) was assessed using the generic, preference-based Short-Form 6D (SF-6D) utility index. The SF-6D is derived from the Short Form-12 (SF-12), which is a subset of the broader SF-36. Patients completed the Norwegian translation of SF-12 version 1 (ProCore license number: QUO-06032-L8Z4W0). Responses to its eight domains were mapped onto the six SF-6D dimensions: physical functioning, role limitations, social functioning, pain, mental health, and vitality. Utility weights for the SF-6D were derived from a UK general population sample based on standard gamble valuation technique to estimate the SF-6D utility index.^1^ These weights were applied to patient responses with ProCore scoring software to generate utility values. The SF-6D index provides a continuous scale ranging from 0 (worst health state) to 1.00 (full health).^1^ Utility scores, estimated at baseline, 9 weeks, 6 months, and 2 years were used to calculate quality-adjusted life years (QALYs) at 6 months and 2 years using the trapezoid rule. No baseline adjustment was performed for QALYs since the participants SF-6D index scores at baseline were similar.

##

## 2. Estimating Costs

### **2.1 Valuing costs**

Costs were valued using the following per unit prices.

##### **Table S5. Unit costs in 2019 Euros**

| **Cost type** | **Cost (€)** | **Unit** | **Source** |
| --- | --- | --- | --- |
| Programme Cost of dCBT-I | 70.16 | per patient | Lump sum fee, 75% in agreement with collaboration partner |
| Programme Cost of PE | 23.80 | per patient | Lump sum fee, 25% in agreement with collaboration partner |
| Sleep medicine | 0.29 | per pill | Norwegian medicines agency maximum retail price:  Zopiclone 7.5 mg 100-pack^1^ |
| GP | 52.57 | per visit | Norwegian medicines agency cost per GP specialist visit^2^ |
| Psychiatrist | 71.25 | per visit | Norwegian medicines agency outpatient cost per specialist visit^3^ |
| Outpatient specialist | 71.25 | per visit | Norwegian medicines agency outpatient cost per specialist visit^3^ |
| Absenteeism cost | 33.32 | per hour | National mean wage per hour from Statistics Norway^4^ |
| Presenteeism cost | 33.32 | per hour | National mean wage per hour from Statistics Norway^4^ |
| GP (patient copay) | 19.22 | per visit | Health Norway (HelseNorge)^5^ |
| Psychiatrist (patient copay) | 32.96 | per visit | Health Norway (HelseNorge)^5^ |
| Outpatient specialist (patient copay) | 32.96 | per visit | Health Norway (HelseNorge)^5^ |

### **2.2 Calculation of costs**

Costs were measured in terms of programme costs, medical costs, out-of-pocket costs and productivity costs. The societal perspective (base case) included all cost types, except for patient out of pocket expenses since these were accounted for in the medical costs. Cost types included in the additional perspectives is detailed in Table S6:

##### **Table S6. Costs in various perspectives**

|  | **Perspectives** | | | |
| --- | --- | --- | --- | --- |
| **Cost type** | Societal | Health Sector | Employer | Patient |
| Programme | X | X | X | X |
| Medical  GP  Psychiatrist  Other specialist  Sleep Medicine | X | X |  |  |
| Productivity  Absenteeism  Presenteeism | X |  | X |  |
| Out of pocket  GP - copay  Psychiatirst - copay  Other specialist - copay  Sleep Medicine |  |  |  | X |

To illustrate the effect of missing data on estimating cumulative costs by category and perspective, Table S7 summarizes total costs for 6 hypothetical patients with varying missing cost components. This example shows the mean societal cost or health sector costs per patient do not equal the sum of the mean cost components.

##### **Table S7. Illustrative example of estimating cost by category and by perspective**

|  | **Cost component** | | | | |  | **Total Cost by Category** | |  | **Total Cost by Perspective** | |
| --- | --- | --- | --- | --- | --- | --- | --- | --- | --- | --- | --- |
| ID | Program | GP | SleepMed | Pres | Abs |  | Medical | Productivity |  | Societal | Health Sector |
|  |  |  |  |  |  |  | *GP+SleepMed* | *Pres+Abs* |  | *Med+Prod+Prog* | *Med+Prog* |
| 1 | 70 | 10 | 5 | 30 | 30 |  | 15 | 60 |  | 145 | 85 |
| 2 | 70 |  | 5 | 30 | 30 |  | 5 | 60 |  | 135 | 75 |
| 3 | 70 | 10 |  | 30 | 30 |  | 10 | 60 |  | 140 | 80 |
| 4 | 70 | 10 | 5 |  | 30 |  | 15 | 30 |  | 115 | 85 |
| 5 | 70 | 10 | 5 |  |  |  | 15 | 0 |  | 85 | 85 |
| 6 | 70 |  |  | 30 | 30 |  | 0 | 60 |  | 130 | 70 |
| Mean | 70 | 10 | 5 | 30 | 30 |  | 11 | 53 |  | 134 | 81 |

SleepMed: Sleep medication; Pres: Presenteeism; Abs: Absenteeism; Med: Medical; Prod: Productivity.

####

#### **Programme costs**

Following the checklist by Khan et al (2024),^6^ we estimated program costs in terms of development and maintenance. A lump sum license fee of NOK 800,000 (€81,196) for the present study was paid to the collaboration partner, The University of Virginia. This covered programme costs of both SHUTi and PE for the study’s participants. In agreement with the collaboration partner, we assumed that 75% of the license fee could be attributed to SHUTi, whereas 25% was attributed to PE. The fee was divided by the number of participants in each group to obtain per-participant programme costs for SHUTi and PE respectively.

####

#### **Medical Costs**

Medical costs included GP visits, outpatient clinical visits and sleep medication use. Patients reported health service use at baseline, 6 months and 2 years in terms of “yes” or “no” responses. They were asked if they had an outpatient visit to a GP, psychiatrist or another specialist in the past 3 months. In order to estimate volume of visits and based on expert opinions, we assumed that each “yes” response corresponded to one visit per quarter to the GP, and one visit per 6 months to the psychiatrist or specialist. At baseline, costs were estimated for the succeeding 9 weeks based on the patients’ baseline self-reports. Thereafter, patients’ responses to the 6 months and 2 years questionnaires were used to extrapolate number of visits post-treatment for the respective time periods. As shown in Table S5, number of visits were valued using 2019 cost per visit estimates from Norwegian Medical Products Agency.^2,3^

Patients reported sleep medicine use in consensus diaries at baseline, 9 weeks, 6 months and 2 years, as daily “yes” or “no” responses for a maximum of 14 days. We estimated sleep medicine use as a ratio between the total number of sleep medicine days and the total number of diary days per patient. This ratio was assumed to represent daily sleep medicine use for the patient. For example, a patient indicating 6 days of sleep medicine use in 6 days of diary completion was assumed to be taking 1 pill daily, whereas a patient reporting sleep medicine use in 3 out of 6 days of diary completion was assumed to be taking 0.5 pills daily. The daily dose ratios reported at baseline were extrapolated to cover the 9 weeks of treatment. Costs at 6 months follow-up were estimated based on daily dose ratio at 6 months report. If this value was missing, patients’ report of daily dose ratios from 9 weeks were carried forward. Costs at 2 years follow up were estimated estimated based on patients report at 2 years. We visually inspected patient’s free text responses of medicine use, and after consultation with an expert, for estimation purposes we assumed that patients indicating sleep medicine use were taking Zopiclone 7.5 mg per day. We applied the Norwegian medicines agency maximum retail price per pill of Zopiclone 7.5 mg in 100-pack to estimate the sleep medicine costs per patient (Table S5).

#### **Out of pocket costs**

Patients in Norway make a co-payment for outpatient GP or specialist visits, which are capped to an annual limit by the government.^5^ We estimated patients’ out of pocket expenses based on the estimated volume of health service use (i.e. GP visits, outpatient psychiatrist visits, specialist visits and sleep medicine use). We valued the patients cost per visit by applying the standard copay for a visit to a GP or specialist in the Norwegian public health system based on information from Health Norway (*HelseNorge*).^5,7^ Since sleep medicine is usually purchased over-the-counter, and not covered by the public health system, we assumed that the all sleep medication costs were out-of-pocket. Out-of-pocket costs over the 2-year time horizon are summarized in Table S8.

##### **Table S8. Patients’ out-of-pocket medical costs at 2 years.**

|  |  | **dCBTi** | |  | **PE** | |  | **Incremental** |  |
| --- | --- | --- | --- | --- | --- | --- | --- | --- | --- |
|  |  | **n** | **Mean (95% CI)** |  | **n** | **Mean (95% CI)** |  | **Mean (95% CI)** | **p** |
| Patient out of pocket medical costs | |  |  |  |  |  |  |  |  |
|  | GP copay | 310 | 82 (79 to 86) |  | 268 | 99 (95 to 103) |  | -17 (-22 to -12) | 0.00 |
|  | Psychiatrist copay | 310 | 9 (7 to 10) |  | 268 | 7 (5 to 8) |  | 2 (0 to 4) | 0.09 |
|  | Other specialist copay | 308 | 21 (18 to 23) |  | 268 | 24 (22 to 27) |  | -4 (-7 to 0) | 0.03 |
|  | Sleep medication | 263 | 41 (38 to 45) |  | 224 | 53 (50 to 74) |  | -12 (-16 to -7) | 0.00 |
| Sum of patients' out of pocket 'costs | | 868 | 146 (140 to 153) |  | 853 | 175 (168 to 181) |  | -28 (-37 to -19) | 0.00 |

#### **Productivity Costs**

Productivity losses in terms of reduced productivity at work and absenteeism caused by insomnia were estimated using the Work Productivity and Activity Impairment (WPAI) questionnaire. The WPAI is a 6-item instrument used to assess the impact of health problems on work productivity and daily activities.^8^ Following the approach by Kyle et al., we quantified productivity losses using questions 1, 2, 4, 5 and 6 of WPAI, and summarized them as absenteeism and presenteeism hours for those employed (n = 1321) and activity impairment in daily activities for the whole sample (n=1721).^9^ Productivity costs were estimated based on hours of presenteeism and absenteeism. Percentage of activity impairment was reported as a secondary outcome.

WPAI was scored according to the guidance by Reilly Associates.^8^ Patients reported their current employment status (Q1) and hours worked per week (Q4) at baseline, which were assumed to remain unchanged at subsequent time points (i.e. 6 months and 2 years). Patients reported hours missed due to health problems in the past 4 weeks (Q2). We estimated weekly absenteeism hours by firstly dividing Q2 responses with the sum of hours worked per month (Q4) and hours missed per month (Q2), and next multiplying the resulting proportion with hours worked per week (Q4). Patients reported productivity loss while at work due to health problems (Q5) in the past week on a 0 to 10 scale. We estimated weekly presenteeism hours by dividing Q5 responses by 10 and multiplying the resulting proportion with hours worked per week (Q4). Patients reported activity impairment in regular non-work activities (Q6) on a 0 to 10 scale, which was converted into a percentage by dividing patients’ responses by 10.

For consistency, weekly absenteeism and presenteeism hours were converted into monthly hours by applying a multiplier of 4, assuming each month has 4 weeks. Productivity losses at 6 months were estimated by multiplying the estimated monthly hours of absenteeism and presenteeism hours by 6. Similarly, productivity losses at 2-year were estimated by multiplying the estimated monthly hours of absenteeism and presenteeism hours by 24. For patients that reported at both 6 months and 2 years timepoints, 2-year productivity losses were estimated by applying a multiplier of 18, i.e. 2 years minus 6 months. Patients’ reported absenteeism and presenteeism at baseline were extrapolated to cover the 9 weeks of treatment, whereas the costs estimated at 6 months and 2 years covered the follow up period. As shown in Table S5 productivity losses were valued by multiplying hours lost by average hourly salaries based on median earnings obtained from Statistics Norway 2019.^4^

## 3. Sensitivity Analyses

## 3.1 Sensitivity Analysis 1: Varying Perspectives

We extended the base case societal perspective to include cost-effectiveness estimates from health care sector, employer and patient perspectives. The incremental QALY gain of 0.025 (95% CI: 0.010 to 0.041) experienced by the dCBTI group compared to PE was equal across different cost perspectives. The specific costs included in different perspectives are described in Table S6. Results showed that the incremental cost per patient were similar for both societal and employer perspectives (-€278 [95% CI: -1413 to 858] and -€273 [95% CI: -1703 to 1158] ) with bootstrap analyses indicating respective 96.7% and 99.7% likelihood that ICERs fall below the chosen willingness to pay threshold. In the health sector and patient perspectives, the dCBTI group’s incremental costs were €41 [95% CI: 26 to 56] and €44 [95% CI: 37 to 51], with 96.7% and 98.8% likelihood that the ICERs fall below the willingness to pay threshold. Table S9 summarizes the results.

##### **Table S9. Incremental costs and QALYs in societal, healthcare sector, employer and patient perspectives at 2-years**

| **Perspectives** | **Incremental Costs** | **Incremental QALYs** | **ICER** | **% < WTP ^a^** |
| --- | --- | --- | --- | --- |
| Societal | -278 (-1413 to 858) | 0.025 (0.01 to 0.041) | -10973 | 96.7 |
| Healthcare sector | 41 (26 to 56) | 0.025 (0.01 to 0.041) | 1629 | 99.7 |
| Employer | -273 (-1703 to 1158) | 0.025 (0.01 to 0.041) | -10771 | 96.7 |
| Patient | 44 (37 to 51) | 0.025 (0.01 to 0.041) | 1728 | 98.8 |

^a^ From1000 bootstrap replications

## 3.2 Sensitivity Analysis 2: Varying Time Horizon

We shortened the time horizon to 6 months to assess if the intervention would have been cost-effective with a shorter follow up period. At 6 months follow up, the dCBTI group experienced an incremental QALY gain of 0.010 (0.003 to 0.015) compared to PE. The incremental cost per patient were similar for both societal and employer perspectives (-€317 [95% CI: -806 to 171]) and -€316 [95% CI: -828 to 197])) with bootstrap analyses indicating respective 99.8% and 99.7% likelihood that ICERs fall below the willingness-to-pay threshold. In the health sector and patient perspectives, the dCBTI group’s incremental costs were €45 [95% CI: 40 to 49] and €44 [95% CI: 42 to 46] respectively, with 99.7% likelihood that the ICERs fall below the willingness-to-pay threshold. Shortening the time horizon did not substantially change the incremental costs, but it reduced the incremental QALYs. However, the intervention remained cost-effective in all analytical perspectives.

##### **Table S10. Incremental costs and QALYs in societal, healthcare sector, employer and patient perspectives at 6-months**

| **Perspectives** | **Incremental Costs** | **Incremental QALYs** | **ICER** | **% < WTP ^a^** |
| --- | --- | --- | --- | --- |
| Societal | -317 (-806 to 171) | 0.01 (0.003 to 0.015) | -32616 | 99.8 |
| Healthcare sector | 45 (40 to 49) | 0.01 (0.003 to 0.015) | 4861.2 | 99.7 |
| Employer | -324 (-796 to 148) | 0.01 (0.003 to 0.015) | -34354 | 99.7 |
| Patient | 29 (27 to 31) | 0.01 (0.003 to 0.015) | 4551 | 99.7 |

^a^ From 1000 bootstrap replications

## 3.3 Sensitivity Analysis 3: Varying costs – One-way deterministic

We tested the robustness of our base case estimates in the societal perspective by increasing the program cost and medical cost, and reducing productivity losses of dCBTI group. We increased the program costs per patient up till a maximum given still being cost-effective (see below), and we increased the medical costs and productivity costs (reducing loss) by up till 50%. Since we only changed costs in the intervention group, the resulting ICERs are more conservative than our base case analysis.

dCBTI remained cost-effective when increasing the programme costs from €70.16, until a maximum program cost of €1,110 per patient. Increasing the medical costs by 50% increased the ICER from –€10,973 to –€8,669. Decreasing productivity loss by 20% increased the ICER from –€10,973 to €36,403. In the latter case, SHUTi is no longer considered cost-effective compared to a willingness-to-pay threshold of €30,000.

##### **Table S11. Change in incremental cost-effectiveness ratio (ICER) relative to change in programme costs, medical costs and productivity costs of dCBT-I**

| **Program cost (€)** | **ICER Societal** | **ICER Healthcare** |  | **Medical costs of (€)** | **ICER Societal** | **ICER Healthcare** |  | **Productivity costs (€)** | **ICER Societal** | **ICER Employer** |
| --- | --- | --- | --- | --- | --- | --- | --- | --- | --- | --- |
| Base case | -10,973 | 1,629 |  | Base case | -10,973 | 1,629 |  | Base case | -10,973 | -10,771 |
| 100 | -9,794 | 2,863 |  | + 10% | -10,518 | 2,084 |  | + 10% | 12,715 | 12,917 |
| 348 | 0 | 12,602 |  | + 20% | -10,063 | 2,538 |  | + 20% | 36,403 | 36,605 |
| 789 | 17,418 | 30,020 |  | + 30% | -9,609 | 2,993 |  | + 30% | 60,091 | 60,293 |
| 1,000 | 25,807 | 38,353 |  | + 40% | -9,154 | 3,448 |  | + 40% | 83,779 | 83,981 |
| 1,110 | 30,096 | 42,698 |  | + 50% | -8,699 | 3,903 |  | + 50% | 107,467 | 107,670 |

## 3.4 Sensitivity Analysis 4: Imputing missing data

We imputed missing data on SF-6D, health service utilisation, and productivity losses in accordance with the guidelines by Faria et al. (2014).^10^ Fifty imputed datasets were generated using multiple imputation (MI) with chained equations in wide format, using predictive mean matching with the five nearest neighbours. All imputations were conducted at a disaggregated level, after which new variables for QALYs and aggregated costs were generated using the same estimation methods as in the base case. SF-6D index values were imputed at all time-points to derive QALYs. For health service use, we imputed binary indicators for GP, psychiatrist, and other specialist visits, as well as the sleep medication use ratios from the sleep diaries. Productivity losses were estimated by imputing monthly hours of absenteeism and presenteeism. Because productivity costs were calculated only for employed individuals, imputation for these outcomes was performed separately and later merged with the full imputed dataset.

We first assessed the correlation between SF-6D completeness with key demographic and symptom severity variables. In Table S12, we describe the missing data in relation to various demographic characteristics as well as symptom related indicators over time. Overall, attrition was balanced between the groups. Among demographic variables, missing data was associated with age and gender. Among symptom related indicators, missing data was associated with ISI, BIS and Fatigue scores at baseline and 9 weeks, with HADS and SF-6D scores at baseline.

##### **Table S12. Association of selected demographic characteristics and symptom scores with SF-6D missing data at 2 years**

|  |  | **dCBT-I** | | | |  | **PE** | | | |  |  | |
| --- | --- | --- | --- | --- | --- | --- | --- | --- | --- | --- | --- | --- | --- |
|  |  | Completer | | Non-completer | |  | Completer | | Non-completer | |  |  |  |
|  |  | Mean /n | sd /% | Mean /n | sd /% |  | Mean /n | sd /% | Mean /n | sd /% |  | P-value | |
| Baseline N | | 868 | | | |  | 853 | | | |  |  |  |
| Completed 2 years assessment | | 312 | 36% | 556 | 64% |  | 270 | 32% | 583 | 68% |  |  |  |
|  |  |  |  |  |  |  |  |  |  |  |  |  |  |
| Demographic characteristics | |  |  |  |  |  |  |  |  |  |  |  |  |
|  | Age | 42.62 | 14.08 | 45.08 | 13.91 |  | 42.30 | 13.35 | 45.74 | 13.91 |  | 0.491 | ^a^ |
|  | Female | 210 | 67% | 439 | 79% |  | 193 | 71% | 410 | 70% |  | 0.004 | ^b^ |
|  | Married/Cohabiting | 202 | 65% | 337 | 61% |  | 168 | 62% | 367 | 63% |  | 0.336 | ^b^ |
|  | Years of education | 16.65 | 2.96 | 16.27 | 3.10 |  | 16.24 | 2.68 | 16.31 | 3.06 |  | 0.136 | ^a^ |
|  | Children living in the household | 119 | 38% | 184 | 33% |  | 103 | 38% | 214 | 37% |  | 0.453 | ^b^ |
|  | Mobile use (hours per day) | 2.57 | 2.59 | 2.46 | 2.50 |  | 2.69 | 2.64 | 2.47 | 2.59 |  | 0.656 | ^a^ |
|  |  |  |  |  |  |  |  |  |  |  |  |  |  |
| ISI score | |  |  |  |  |  |  |  |  |  |  |  |  |
|  | Baseline | 18.66 | 3.71 | 19.49 | 3.92 |  | 19.79 | 3.93 | 19.54 | 3.98 |  | 0.007 | ^a^ |
|  | 9 weeks | 9.48 | 5.67 | 11.07 | 6.57 |  | 15.58 | 5.30 | 15.03 | 5.24 |  | 0.003 | ^a^ |
|  | 6 months | 10.20 | 5.69 | 10.20 | 6.73 |  | 14.43 | 5.77 | 13.85 | 5.56 |  | 0.513 | ^a^ |
|  | 2 years | 10.78 | 5.89 | 5.33 | 3.21 |  | 13.40 | 5.84 | 7.00 | 5.66 |  | 0.859 | ^a^ |
|  |  |  |  |  |  |  |  |  |  |  |  |  |  |
| BIS | |  |  |  |  |  |  |  |  |  |  |  |  |
|  | Baseline | 26.93 | 7.42 | 28.43 | 7.35 |  | 28.03 | 7.42 | 27.97 | 7.69 |  | 0.042 | ^a^ |
|  | 9 weeks | 13.27 | 9.18 | 15.85 | 10.05 |  | 22.18 | 9.47 | 21.34 | 9.31 |  | 0.004 | ^a^ |
|  | 6 months | 15.10 | 9.42 | 15.16 | 10.56 |  | 21.02 | 9.81 | 19.08 | 9.46 |  | 0.163 | ^a^ |
|  |  |  |  |  |  |  |  |  |  |  |  |  |  |
| Fatigue | |  |  |  |  |  |  |  |  |  |  |  |  |
|  | Baseline | 20.2 | 6.1 | 21.2 | 5.9 |  | 21.4 | 6.0 | 20.8 | 6.0 |  | 0.010 | ^a^ |
|  | 9 weeks | 14.4 | 6.7 | 16.2 | 7.5 |  | 17.8 | 6.6 | 17.6 | 6.3 |  | 0.019 | ^a^ |
|  | 6 months | 15.7 | 6.4 | 15.7 | 7.2 |  | 17.4 | 6.3 | 17.4 | 6.3 |  | 0.961 | ^a^ |
|  |  |  |  |  |  |  |  |  |  |  |  |  |  |
| HADS | |  |  |  |  |  |  |  |  |  |  |  |  |
|  | Baseline | 13.00 | 6.58 | 13.36 | 7.09 |  | 14.42 | 6.79 | 12.94 | 7.30 |  | 0.010 | ^a^ |
|  | 9 weeks | 9.81 | 6.53 | 10.55 | 7.26 |  | 11.92 | 7.07 | 11.21 | 6.90 |  | 0.097 | ^a^ |
|  | 6 months | 10.01 | 6.74 | 9.88 | 7.07 |  | 11.20 | 6.85 | 10.36 | 6.81 |  | 0.495 | ^a^ |
|  |  |  |  |  |  |  |  |  |  |  |  |  |  |
| SF-6D | |  |  |  |  |  |  |  |  |  |  |  |  |
|  | Baseline | 0.59 | 0.08 | 0.58 | 0.08 |  | 0.58 | 0.08 | 0.59 | 0.08 |  | 0.039 | ^a^ |
|  | 9 weeks | 0.63 | 0.09 | 0.62 | 0.08 |  | 0.60 | 0.08 | 0.61 | 0.08 |  | 0.077 | ^a^ |
|  | 6 months | 0.62 | 0.08 | 0.62 | 0.09 |  | 0.61 | 0.08 | 0.61 | 0.08 |  | 0.624 | ^a^ |

^a^ Generalized linear model; ^b^ Logistic regression. P-values represent a test of the interaction between missingness on SF-6D and group on the listed outcome variables.

Based on these associations, the imputation model included ISI, BIS, fatigue, and HADS scores at all time points, along with age, gender, education, marital status, and whether the respondent had children at home. In the merged imputed dataset, mean QALYs and costs were estimated using linear regression with baseline adjustment. Parameter estimates were combined using Rubin’s rules.

Following imputation, medical costs for the dCBT-I and PE groups remained consistent with the base case, yielding similar incremental costs (MI: –€64 [95% CI: –€91 to –€36] vs. Base case: –€60 [95% CI: –€80 to –€41]). However, mean absenteeism costs in the dCBT-I group increased from €2,794 in the base case to €3,595 post-imputation, reducing the incremental cost difference from –€457 [95% CI: –€1,147 to €232] to –€60 [95% CI: –€1,031 to €911]. Presenteeism costs also increased in the dCBT-I group from base case €21,847 to MI €23,483, while decreasing in the PE group from base case €26,519 to MI €25,395, resulting in a smaller incremental cost (–€1,926) than in the base case (–€4,934). Although total and incremental costs from the health sector perspective remained comparable to the base case, societal costs differed more substantially (MI: –€2,092 vs. Base case: –€278), largely due to the inclusion of a greater number of participants with non-missing productivity data. Post-imputation, the dCBT-I group gained 0.013 QALYs [95% CI: –0.002 to 0.029] relative to PE, which was lower than the base case estimate of 0.025 [95% CI: 0.010 to 0.041]. Nevertheless, the intervention appeared more favourable in the societal and health sector ICERs, primarily due to changes in incremental costs.

#####

##### **Table S13. Multiply imputed costs and QALYs in societal and healthcare sector perspectives at 2-years**

|  |  | **dCBTi** | |  | **PE** | |  | **Incremental ^a^** |
| --- | --- | --- | --- | --- | --- | --- | --- | --- |
|  |  | **n** | **Mean (95% CI)** |  | **n** | **Mean (95% CI)** |  | **Mean (95% CI)** |
| Intervention costs | |  |  |  |  |  |  |  |
|  | Program costs | 868 | 70.16 |  | 853 | 23.80 |  | 46.36 |
| Medical costs | |  |  |  |  |  |  |  |
|  | GP | 868 | 232 (216 to 248) |  | 853 | 270 (255 to 285) |  | -39 (-59 to -19) |
|  | Psychiatrist outpatient | 868 | 19 (14 to 25) |  | 853 | 19 (13 to 26) |  | 1 (-7 to 9) |
|  | Other specialist outpatient | 868 | 46 (38 to 54) |  | 853 | 53 (45 to 61) |  | -8 (-18 to 3) |
|  | Sleep medication | 868 | 48 (43 to 54) |  | 853 | 66 (59 to 72) |  | -14 (-22 to -7) |
| Sum of medical costs | | 868 | 346 (325 to 367) |  | 853 | 408 (386 to 429) |  | -64 (-91 to -36) |
| Productivity costs | |  |  |  |  |  |  |  |
|  | Absenteeism | 662 | 3,595 (2,889 to 4,302) |  | 665 | 3,495 (2,812 to 4,178) |  | -60 (-1,031 to 911) |
|  | Presenteeism | 662 | 23,483 (20,600 to 26,365) |  | 665 | 25,395 (22,819 to 27,970) |  | -1,926 (-5,477 to 1,626) |
| Sum of productivity costs | | 662 | 27,078 (2,3774 to 30,382) |  | 665 | 28,889 (25,976 to 31,803) |  | -2,179 (-6,201 to 1,844) |
| Total costs | | 0 |  |  | 665 | 28889 (25976 to 31803) |  |  |
|  | Societal Perspective | 868 | 21,029 (18,393 to 23,665) |  | 853 | 22,964 (20,552 to 25,376) |  | -2,092 (-5,264 to 1,079) |
|  | Healthcare sector perspective | 868 | 416 (395 to 437) |  | 853 | 431 (410 to 453) |  | -17 (-45 to 10) |
|  |  |  |  |  |  |  |  |  |
| QALYs |  | 868 | 1.343 (1.332 to 1.354 ) |  | 853 | 1.33 (1.319 to 1.341) |  | 0.013 (-0.002 to 0.029 ) |
| ICER |  |  |  |  |  |  |  |  |
|  | Societal Perspective |  |  |  |  |  |  | -158,396 |
|  | Healthcare sector perspective | |  |  |  |  |  | -1,316 |

^a^ Estimate from linear regression with baseline adjustment

## 3.5 Sensitivity Analysis 5: Imputing with missing not at random scenarios

To explore the robustness of the base-case results under plausible missing not at random (MNAR) assumptions, we conducted a series of sensitivity analyses using the pattern mixture modelling (PMM) framework. Specifically, we applied a delta adjustment approach to the imputed values for participants in the dCBTI group, where outcome values were assumed to be systematically worse than under the missing at random (MAR) assumption. Following multiple imputation under MAR, we introduced deterministic offsets by reducing imputed SF-6D values by 5%, 10%, and 20% for individuals in the intervention group only. Similarly, we re-estimated total societal costs by increasing productivity costs and medical costs by 5%, 10% and 20% for individuals in the intervention group only. No adjustments were made for the PE group, consistent with the assumption that differential attrition would more likely bias estimates in favour of the intervention. Adjusted imputed datasets were used to re-calculate QALYs and cost outcomes using the same post-estimation algorithms as the base-case analysis. This approach allowed us to assess how incremental cost-effectiveness ratios (ICERs) would change under increasingly conservative assumptions about missingness in the intervention arm.

Results from these conservative analyses indicated that decreasing QALYs by even 5% in the intervention arm made dCBTI less effective than PE, rendering the intervention not cost-effective. On the other hand, dCBTI remained cost-effective in the societal perspective until 10% increase in costs (ICER: €81 per QALY) but crossed the willingness to pay threshold when costs in the intervention arm increased by 15% (ICER: €79,319 per QALY). In the health sector perspective, dCBTI remained cost-effective even when costs were increased by 20% (ICER: €4,972 per QALY). This underscores the importance of productivity costs in relation to insomnia.

#####

##### **Table S14. Incremental costs and QALYs in societal and health care sector perspectives at 2-years in missing not at random scenarios**

| **Societal Perspective (Incremental Cost; Incremental QALYs)** | | | | |  |  |
| --- | --- | --- | --- | --- | --- | --- |
|  |  | **QALYs** | | | | |
|  |  | **Unchanged** | **-5%** | **-10%** | **-15%** | **-20%** |
| **Costs** | **Unchanged** | -2092; 0.013 ^a^ | -2092; -0.054 | -2092; -0.121 | -2092; -0.188 | -2092; -0.255 |
|  | **+5%** | -1046; 0.013 ^a^ | -1046; -0.054 | -1046; -0.121 | -1046; -0.188 | -1046; -0.255 |
|  | **+10%** | 1.07; 0.013 ^a^ | 1.07; -0.054 | 1.07; -0.121 | 1.07; -0.188 | 1.07; -0.255 |
|  | **+15%** | 1048; 0.013 | 1048; -0.054 | 1048; -0.121 | 1048; -0.188 | 1048; -0.255 |
|  | **+20%** | 2094; 0.013 | 2094; -0.054 | 2094; -0.121 | 2094; -0.188 | 2094; -0.255 |
|  |  |  |  |  |  |  |
| **Health Sector Perspective (Incremental Cost; Incremental QALYs)** | | | | | | |
|  |  | **QALYs** | | | | |
|  |  | **Unchanged** | **-5%** | **-10%** | **-15%** | **-20%** |
| **Costs** | **Unchanged** | -17; 0.013 ^a^ | -17; -0.054 | -17; -0.121 | -17; -0.188 | -17; -0.255 |
|  | **+5%** | 3.38; 0.013 ^a^ | 3.38; -0.054 | 3.38; -0.121 | 3.38; -0.188 | 3.38; -0.255 |
|  | **+10%** | 24; 0.013 ^a^ | 24; -0.054 | 24; -0.121 | 24; -0.188 | 24; -0.255 |
|  | **+15%** | 45; 0.013 ^a^ | 45; -0.054 | 45; -0.121 | 45; -0.188 | 45; -0.255 |
|  | **+20%** | 66; 0.013 ^a^ | 66; -0.054 | 66; -0.121 | 66; -0.188 | 66; -0.255 |

^a^ ICER below willingness to pay threshold.

# Appendix C: Secondary outcomes listed in the trial protocol that are not reported here

| **Outcomes** | **6 months follow-up** | **2 years follow-up** |
| --- | --- | --- |
| Marital status | x | x |
| Body mass index | x | x |
| Number of children living at home | x | x |
| Brief Morningness-Eveningness Questionnaire | x | x |
| Brief Dysfunctional Beliefs and Attitudes Scale 16 | x | x |
| Other pain, physical health, physical activity, and mental health symptoms (Adapted from the Nord-Trøndelag Health Study) | x | x |
| Alcohol Use Disorders Identification Test- Consumption | x | x |
| Electronic media use | x | x |
| Internet intervention evaluation |  | x |
| Long-term use of sleep-strategies |  | x |
| Negative effects of treatment |  | x |

**References**

1. Norwegian Medicines Agency. Maximum retail price: Zopiclone 7.5 mg 100-pack 2023 [Available from: <https://www.dmp.no/en/public-funding-and-pricing/pricing-of-medicines/maximum-price>.

2. Norwegian Medicines Agency. Cost for GP specialist visit. Unit Cost database2023.

3. Norwegian Medicines Agency. Cost for outpatient specialist visit. Unit Cost database2023.

4. Statistics Norway. Table 11419: Monthly earnings, by measuring method, occupation, sector, industry (SIC2007), sex, contents, year and contractual/usual working hours per week. Web: Statistics Norway; 2023. p. 2022 Mean earnings for 84.11 General public administation activities, both genders, full and part time employees.

5. Norsk helsenett. User fees at the family doctor in Norway 2020 [Available from: <https://www.helsenorge.no/en/payment-for-health-services/user-fees-at-the-family-doctor/>.

6. Khan ZA, Kidholm K, Pedersen SA, Haga SM, Drozd F, Sundrehagen T, et al. Developing a Program Costs Checklist of Digital Health Interventions: A Scoping Review and Empirical Case Study. Pharmacoeconomics. 2024;42(6):663-78.

7. Norsk helsenett. About Helsenorge Oslo: The Norwegian Directorate of Health,; 2023 [Available from: <https://www.helsenorge.no/en/about-helsenorge/>.

8. Associates R. WPAI Scoring 2024 [Available from: <http://www.reillyassociates.net/wpai_scoring.html>

9. Kyle SD, Siriwardena AN, Espie CA, Yang Y, Petrou S, Ogburn E, et al. Clinical and cost-effectiveness of nurse-delivered sleep restriction therapy for insomnia in primary care (HABIT): a pragmatic, superiority, open-label, randomised controlled trial. The Lancet. 2023;402(10406):975-87.

10. Faria R, Gomes M, Epstein D, White IR. A Guide to Handling Missing Data in Cost-Effectiveness Analysis Conducted Within Randomised Controlled Trials. PharmacoEconomics. 2014;32(12):1157-70.
